# Flexible Ultra-Thin Nanocomposite Based Piezoresistive Pressure Sensors for Foot Pressure Distribution Measurement

**DOI:** 10.3390/s21186082

**Published:** 2021-09-10

**Authors:** Dhivakar Rajendran, Rajarajan Ramalingame, Saravanan Palaniyappan, Guntram Wagner, Olfa Kanoun

**Affiliations:** 1 Measurement and Sensor Technology, Technische Universität Chemnitz, Reichenhainer Straße 70, 09126 Chemnitz, Germany; dhivakar.rajendran@etit.tu-chemnitz.de (D.R.); rajarajan.ramalingame@etit.tu-chemnitz.de (R.R.); 2 Composites and Material Compounds, Institute of Material Science and Engineering (IWW), Technische Universität Chemnitz, Erfenschlager Straße 73, 09125 Chemnitz, Germany; saravanan.palaniyappan@mb.tu-chemnitz.de (S.P.); guntram.wagner@mb.tu-chemnitz.de (G.W.)

**Keywords:** foot pressure distribution, gait analysis, flexible sensors, wearable sensors, multi carbon nanotubes (MWCNT), polydimethylsiloxane (PDMS), pressure sensor, bio-medical applications, nanocomposite sensors

## Abstract

Foot pressure measurement plays an essential role in healthcare applications, clinical rehabilitation, sports training and pedestrian navigation. Among various foot pressure measurement techniques, in-shoe sensors are flexible and can measure the pressure distribution accurately. In this paper, we describe the design and characterization of flexible and low-cost multi-walled carbon nanotubes (MWCNT)/Polydimethylsiloxane (PDMS) based pressure sensors for foot pressure monitoring. The sensors have excellent electrical and mechanical properties an show a stable response at constant pressure loadings for over 5000 cycles. They have a high sensitivity of 4.4 kΩ/kPa and the hysteresis effect corresponds to an energy loss of less than 1.7%. The measurement deviation is of maximally 0.13% relative to the maximal relative resistance. The sensors have a measurement range of up to 330 kPa. The experimental investigations show that the sensors have repeatable responses at different pressure loading rates (5 N/s to 50 N/s). In this paper, we focus on the demonstration of the functionality of an in-sole based on MWCNT/PDMS nanocomposite pressure sensors, weighing approx. 9.46 g, by investigating the foot pressure distribution while walking and standing. The foot pressure distribution was investigated by measuring the resistance changes of the pressure sensors for a person while walking and standing. The results show that pressure distribution is higher in the forefoot and the heel while standing in a normal position. The foot pressure distribution is transferred from the heel to the entire foot and further transferred to the forefoot during the first instance of the gait cycle.

## 1. Introduction

The International Diabetes Federation (IDF) has estimated that there are currently around 59.3 million people with diabetes in Europe. Among them, around 1.1 million people suffered from diabetic foot ulcers each year [1]. Several studies show that the sudden change in unbalanced gait and foot complications in diabetes can be related to initial symptoms of diabetic foot ulcers (DFU), which can be reduced by up to 85% if detected at an early stage [2,3]. In sports, it was reported that the psychology of a sportsperson could be related to the unbalanced gait during the sports activities [4]. Monitoring gait can be, therefore, helping to identify the mentality of the people during their sports activities and reduce the sport-related injuries, where 59% people were subjected around the world [4]. Similarly, it is reported that patients with temporomandibular joints (TMJ) are associated with poor balance and posture [5]. During treatment, It is essential to monitor the disturbance in posture in order to exploit the potential of occlusion splint for TMJ disorders [5,6]. So, foot pressure distributions analysis has become one of the necessary research works on sports and related biomedical applications to reveal the interfacial pressure between the foot plantar surface and ground, which helps in diagnosis and rehabilitation of foot problems, injury prevention, sports performance analysis and improves balance control [4,7].

In the 21st century, various low-cost conventional techniques such as microfluidic using silicone layer [8], air pressure using air bladder [8], and optical fibers [9] are used for measuring foot pressure in both static and dynamic mode, and it can reach pressure range up to 150 kPa. However, these techniques are not suitable for large deformations and fail to cover a wide pressure range. They are not durable and not comfortable for dynamic load applications [10]. The introduction of electrical sensors paved the way for implementing the in-shoe-based foot pressure measurement method. In-shoe sensors are flexible and embedded in the commercial shoes so that they can measure the interface between the foot and the shoe, which gives more comfort than platform methods [10]. Several commercial foot pressure measurement products such as PEDAR, TEKSCAN, Medilogic and Orpyx LogR were available in the market [10,11,12,13,14]. These products have different sensors placement, and they reach up to 960 sensors implemented in the shoe with the minimum thickness of 1500 μm. However, the cost of these commercial systems is high.

### Sensors for Foot Pressure Monitoring

To overcome the limitation of the commercial systems, many research attempts have been made to monitor the plantar pressure with novel cost-effective, stretchable, and flexible sensor technology, such as piezoelectric, piezocapacitive and piezoresistive [15]. In piezoelectric sensors, piezoelectric material produces a voltage in response to the force applied. A piezoeelectric insole device based on polyvinylidene fluoride (PVDF) film was fabricated to detect the plantar pressure and harvest energy simultaneously. The total stored energy is around 1.6 pJ by the piezoelectric insoles during an entire walking cycle [16]. A textile-based ferroelectric foot pressure sensor was presented, which is made from fluorinated ethylene propylene (FEP) films, and conventional textile cotton formed to a sandwich structure as foot pressure sensor [17]. Indeed, materials like ZnO, lead zirconate titanate (PZT), MoS2, poly (vinylidene fluoride) (PVDF) and its similar copolymer (PVDF-TAFE) have been used as a piezoelectric material for foot pressure sensors. However, these sensors having a high impedance and are susceptible to electrical interference as they generate signals with a low signal-to-noise ratio [18]. In some investigations, capacitive sensors have been adopted because of their sensitivity and linearity [19,20,21,22]. A capacitive sensor based on the copper layer and flexible PCB to be implemented for foot pressure measurement [19]. Despite its simplicity, it has poor flexibility, which is not good for monitoring foot pressure during sports activities. To enhance the flexibility, PDMS was used as elastomeric dielectric material, gold microsphere composites, CNT/PDMS nanocomposites were used as electrodes, which reaches the pressure range 10–200 kPa [20,21]. Even though it has many advantages, capacitive sensors are vulnerable to external electromagnetic interferences, and humidity reducing their precision [22]. On the other hand, piezoresistive pressure sensors are easy to interface with peripheral circuits, less susceptible to noise and can miniaturize the sensors [23]. Earlier, commercial sensors called force-sensing resistor (FSR) were used in foot pressure measurement systems [24,25,26,27,28], which are ultra-thin, flexible, and have a low power consumption, such as FSR402, LDT0-028K. Investigations show that over time of use, they have been deformed, and their sensitivity was degraded. Therefore, they should be calibrated approximately after every 100 cycles [15]. They reach a low accuracy level with dynamic pressure compared to the static pressure test [15]. A smart sensing system utilizing flexible electromyography (EMG) sensors and a forty eight-channel flexible piezo-resistive sensor. However, a deep investigation of the sensor is still missing [29]. A novel smart insole for monitoring the plantar pressure distribution which has a piezoresistive sensing matrix based on a Velostat layer for transducing applied pressure into an electric signal. It is reported that Velostat presents numerous shortcomings in terms of accuracy and precision when used as a pressure transducer [30]. A liquid type of strain sensor based on a liquid-impregnated porous elastic rope (LIPER) immersed into a flexible silicone matrix [31]. However, these sensors were implemented only in the heel region, and liquids presented in the LIPER are sensitive to the temperature. Several other works, such as crack-based sensor made of gold and chromium layer of polyimide films [32] and laser-induced graphene (LIG) [33] were used as piezoresistive pressure sensors for plantar pressure monitoring, which can monitor up to 120 kPa. These sensors have poor sensitivity, lack of flexibility and a higher thickness of the insole due to multi-layer fabrication [15]. It was reported that a flexible pressure sensor based on micro-structuring of thin films on PDMS substrate could be utilized for biomedical applications. These methods show high sensitivity and fast response time (<10 ms) for minimum pressure of 0.1 Pa. However, the maximum working range of the pressure sensors was 1–7 kPa, which is not be suitable for foot pressure monitoring applications where the initial pressure applied by the foot is approximately >40 kPa [34,35,36,37].

To further reduce the thickness and to enhance the durability of the insole, the development of plantar pressure sensors based on the piezoresistive effect of nanocomposites by incorporating the conductive nanofillers such as carbon nanotubes, graphene in polymers [15]. Nanocomposite based textile sensor are more flexible, and comfortable, when they are based, e.g., on multilayer graphene [38], “E-Textile” [39], carbon nanotubes (CNT)-coated polyester fabric [40], and organic polymers [41], three-layer non-oven textile structures [42], piezoresistive sensor (TPRS) consisting of rG-cotton fabric electrode and Ag fabric [43] with a maximum detectable pressure range of 0.01–1.2 MPa are used in foot pressure measurement applications. Despite its comfort, it failed to achieve durability because textile fibers lost their quality during long term loading conditions. Carbon nanotubes incorporated into polydimethylsiloxane (PDMS) matrix, thereby a pressure sensor on polyimide substrate has been realized for plantar pressure monitoring measuring forces up to 25 N [44]. A combination of mesh-molded CNT/PDMS periodic microstructures for pressure sensing using complex multi-step etching process [45]. An electronic insole was fabricated based on CNT/PDMS pressure sensors with CNT concentration ranges from 0.7 wt. % to 4 wt. %, which can detect a maximum pressure of 250 kPa and implemented to monitor the foot pressure distribution only in the forefoot [44,45,46]. In our previous work, we realized a CNT/PDMS-based pressure sensor for gait analysis by implementing 12 sensors with a sensitivity of 3.3 kΩ/kPa and maximum detectable pressure up to 217 kPa [47].

Based on the reported literature, various sensor technologies have been adopted to realize the foot pressure measurement for different applications. However, the sensor should meet specific requirements to implement it in the foot pressure measurement systems for real-time applications, which is also to be addressed. The main requirements are mobile (weight < 300 g), high sensitivity, wide pressure range, more negligible hysteresis, stable and repeatable. Based on our previous work [47], we develop an optimized fabrication process of nanocomposite pressure sensors consisting of polydimethylsiloxane (PDMS) and multi-walled carbon nanotubes (MWCNT) by using a low concentration of MWCNT (0.3 wt. %) and characterize the electromechanical properties of the pressure sensor to meet the intend requirements for the foot pressure measurement. The developed pressure sensor is evaluated for repeating cycles over 5000 cycles, constant pressure over a time and different rate of pressure loadings (5 N/s to 50 N/s). The developed pressure sensor shows a better response for the above pressure tests, which can be suitable for real-time implementation for the foot pressure measurement. The developed sensors are implemented on the in-sole based foot measurement system (weighing approx. 9.46 g) and the total thickness of 500 μm to monitor the foot pressure distribution while walking and standing.

## 2. Materials and Methods

### 2.1. Preparation of MWCNT/PDMS Nanocomposite Pressure Sensor

Dispersion of multi-walled carbon nanotubes (MWCNT) in PDMS is essential to utilize the full potential of the MWCNT in the polymer [48]. Effective dispersion of MWCNT in PDMS is still a challenging aspect due to the van-der-Waals attraction force between the MWCNT, which tend to bundle and/or reaggregate the MWCNT [48]. In this work, sonication, melt mixing, and solution processing are adapted to use nanocomposites efficiently. Among these, sonication plays a vital role in debundling MWCNT, imparting high shear stress. However, it may reduce the aspect ratio of MWCNT, which should be controlled and monitored. Melt mixing can disperse MWCNT in the matrix homogeneously by providing uniform shear stress. Since PDMS is highly viscous, it is challenging to disperse MWCNT in it homogenously. It can be achieved by a solution processing approach, where an organic solvent is used to disperse in MWCNT later in PDMS. Tetrahydrofuran is selected as the common organic solvent, where it can efficiently disperse the MWCNT in PDMS [49,50]. MWCNT with outer diameter 6–9 nm, an average length of 1 μm and carbon purity of >95% purchased from Sigma-Aldrich is used as conductive nanofillers, PDMS (Sylgard 184 Kit) were purchased from Dow Corning. 0.3 wt. % MWCNT is mixed with tetrahydrofuran (THF) is sonicated using Bandelin Sonopuls HD 3200, then magnetically stirred using CAT M27, followed by mixed with PDMS (1:1).

**Figure 1:**
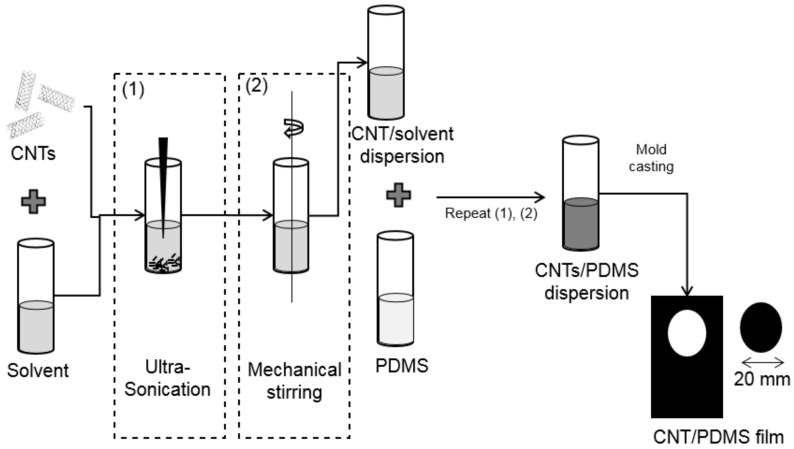
Schematic representation of fabrication of MWCNT/PDMS pressure sensor.

Then, the mixture is sonicated and magnetically stirred once more for better homogeneity. Kapton (HN Polyimide) is used as the substrate because of its superior mechanical properties, which undergo surface pre-treatment with isopropanol to improve the adhesion and remove the impurities on the surface. The electrode is prepared by depositing silver ink on the mask using isolation foil over the substrate and then dried for 15 min at room temperature, and then the mask is peeled off from the substrate. Thin film is prepared using a mold of thickness 600 μm and a radius of 10 mm. Then, it is allowed for curing for 4 h at 120 ${}^{\circ }$C. Peel-off should be done slowly and gently after complete curing, and the final film thickness is 500 µm because of the solvent evaporation. Finally, the sensor has been encapsulated to overcome humidity influence by laminating with PET using Cheminstruments laminator. Figure 1 represents the fabrication of the MWCNT/PDMS pressure sensor.

### 2.2. Piezoresistive Sensing Principle

The electrical conductivity of the MWCNT/PDMS nanocomposite pressure sensor is measured using a digital multimeter Agilent 24401A and can be explained using percolation theory and tunneling effect, where MWCNT act like metal–metal junctions form conduction paths between the electrode and polymer acts like a tunneling barrier between MWCNTs. It can be modeled as three-dimensional resistor networks based on tunneling effect [51]. Theoretically, two types of resistance can be seen in MWCNT–PDMS nanocomposite films such as *R${}_{tube}$* is intrinsic resistance of MWCNT [52]. The second type is *R${}_{junction}$*, which is further divided into contact resistance between MWCNT (*R${}_{C}$*) and tunneling resistance (*R${}_{T}$*) between MWCNT, which can be seen below:

(1) $$R=R_{tube}+R_{junction}$$

(2) $$R_{T}=\frac{h^{2}d}{Ae^{2\sqrt{2m\lambda }}}\times e^{\frac{4\pi d}{h}\sqrt{2m\lambda }}$$

where *d* is the distance between MWCNT, *h* is the Planck constant, *e* is the quantum of electricity, *l* is the barrier height of energy, *m* is the electron mass, and *A* is the cross-sectional area of the tunnel [52]. During the initial applied force of 1 N, the nanocomposite thin film comes in contact with the underlying electrode, resulting in an abrupt decrease in resistance of the sensor. So, the sensor needs an activation force of 1 N to provide a stable measurement. Further increases in force result in further decreases in resistance. It can be explained by the Equations (1) and (2), where a change in orientation of conduction path results in a change of *R${}_{c}$*, change in tunneling distance results in a change of *R${}_{T}$*, which in turn change the junction and deformation of MWCNT change in *R${}_{tube}$*, which results in overall resistance decreases [28].

## 3. Results and Discussion

### 3.1. Material Characterization

**Figure 2:**
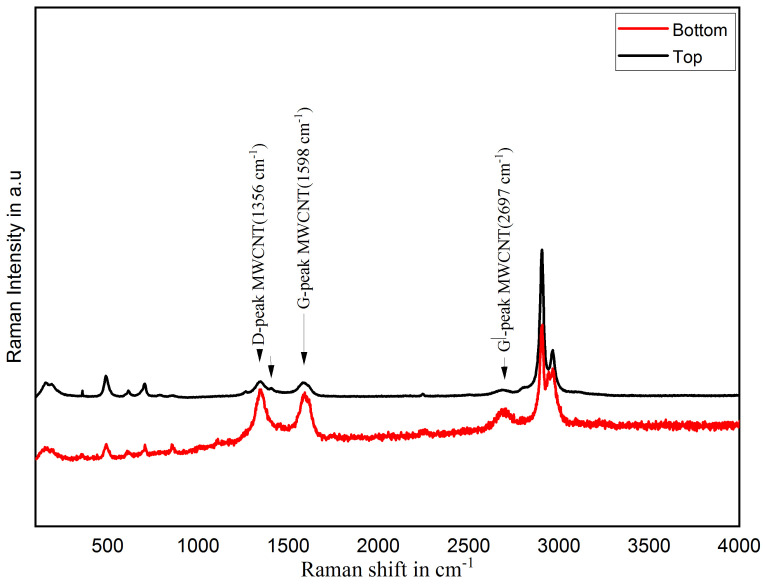
Raman spectra MWCNT/PDMS nanocomposite films.

In this section, material composition is analyzed to the investigated homogenous distribution of MWCNT in PDMS matrix using a non-destructive technique called Raman spectroscopy using Renishaw inVia Raman microscope. MWCNT/PDMS composite films were scanned in extended scan mode for 10 s on the top and bottom side of the cross-section of film using a green laser source with a power of 1 mW and wavelength of 532 nm. Figure 2 shows the Raman spectra of the MWCNT/PDMS nanocomposite films.

**Figure 3:**
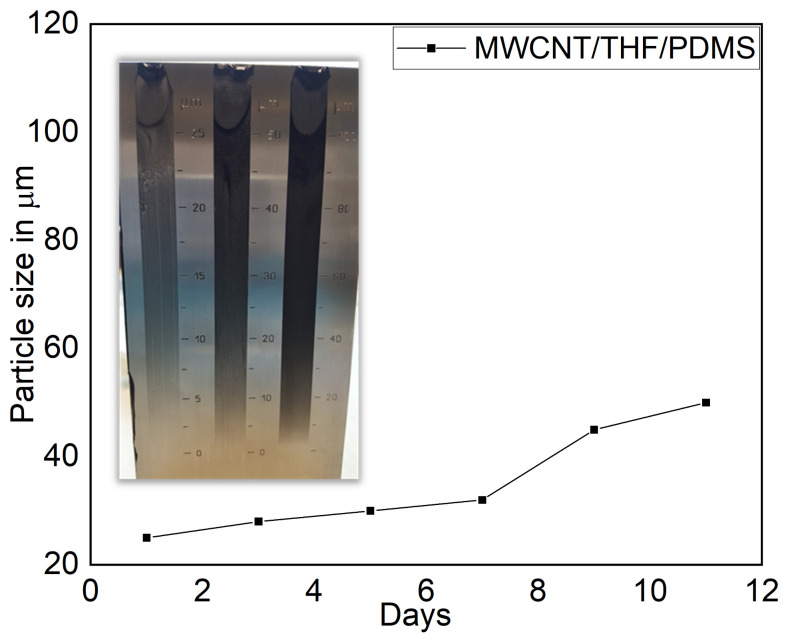
Grindometer test for investigate agglomerates formation.

The D-peak (1356 cm${}^{-1}$) and the G-peak (1598 cm${}^{-1}$) are the characteristic peaks of MWCNT. The other peaks are related to PDMS structure, which is in conformance with characteristic peaks of MWCNT and PDMS [52]. These characteristic peaks can be seen on both surfaces of the thin films, and it shows MWCNT is spread homogenously in the thin films, which may lead to the effective use of MWCNT in nanocomposite sensors. Despite its practical approaches, reaching the homogeneous dispersion of MWCNT in polymer matrices, it is crucial to maintain its stability. It is due to the alternation in binding between MWCNT and polymer over time and MWCNT re-bundle again. Thus, reducing the physical aging rate is essential, as a reliable and longer material lifetime is required for nanocomposite applications. The stability of the MWCNT dispersion state is a valuable indication of the quality of the dispersion, as MWCNT in an unstable environment tends to re-aggregate into bundles over time. The longer the dispersion lasts, the fewer/smaller MWCNT bundles are formed and vice versa. For that, a sample of about 100–500 μL is first applied to the lower end of the wedge-shaped groove of the grindometer to measure the agglomerates size in nanocomposites and then streaked with a slider. Then, the layer thickness is determined with the eye based on the scale at which the paint films look rough due to occurring stripes or punctiform tracks. From Figure 3, MWCNT/PDMS nanocomposite maintains particle size around 20–30 µm for seven days, and it shows nanocomposite is stable for seven days.

### 3.2. Electrical Characterization

**Figure 4:**
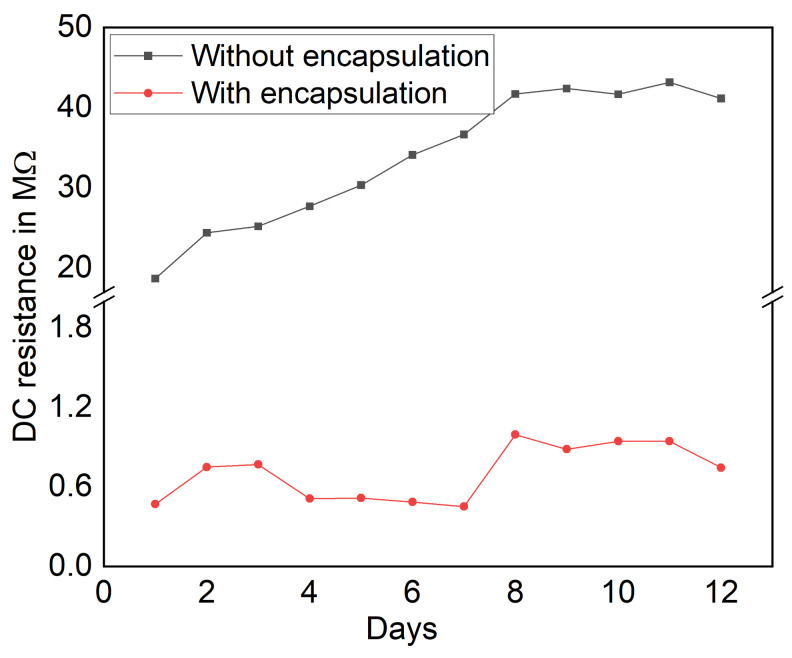
DC resistance of with and without encapsulation of the MWCNT/PDMS pressure sensors over day.

Besides the influence of the polymer and fabrication technique, ambient parameters such as temperature, humidity, and the surrounding conditions might affect nanocomposites’s electrical properties, which should also be considered. Furthermore, the film stability is characterized in terms of DC resistance over days. Figure 4 shows the measured DC resistance of with and without encapsulation of the MWCNT/PDMS pressure sensors over days.

The results show a fluctuation of resistance at the first few days and that the film becomes stable after eight days. In the case of the film without encapsulation, this behavior is mainly influenced by ambient conditions.After encapsulation by laminating with a PET film using a commercial laminator, the initial resistance of the encapsulated sensors is reduced by 90% than non-encapsulated sensor. This is because the encapsulation brings more contact between the underlying nanocomposite films and the electrodes. Compared to the films without encapsulation, the fluctuation in initial resistance is drastically reduced by 93.6% over 12 days.

### 3.3. Electro-Mechanical Characterization

**Figure 5:**
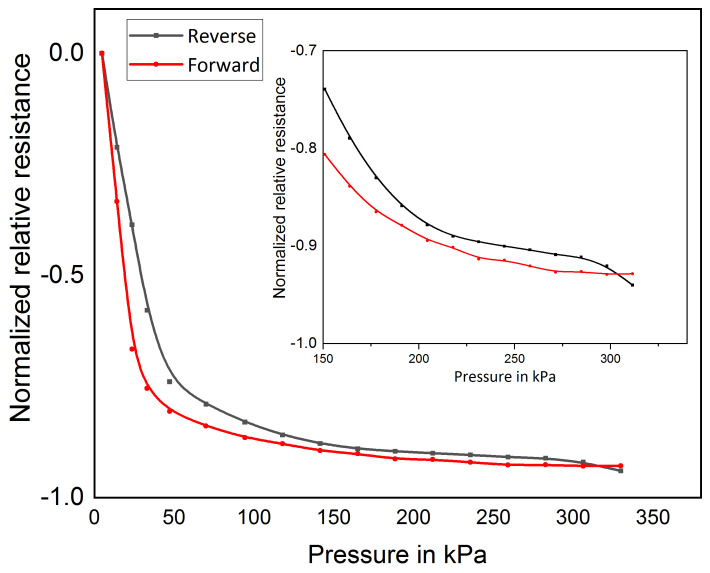
Sensor behavior for forward and reverse force cycle. Inside: Sensor behavior for pressure of 150 to 330 kPa.

In this section, the pressure behavior of thin films based on MWCNT/PDMS nanocomposite is investigated. Using an automated test bench, the thin films subjected to pressure range from 3.1 kPa to 330 kPa for 10 cycles. The selected pressure range (3.1 kPa to 330 kPa) is chosen for the expected foot pressure measurement range. Figure 5 shows the forward and reverse pressure-resistance behavior of the MWCNT/PDMS pressure sensor. The sensor shows a sensitivity of 4.4 kΩ/kPa and a hysteresis corresponding to energy losses of less than 1.7% and a maximal measurement deviation of 0.13% relative to the maximal relative resistance.

**Figure 6:**
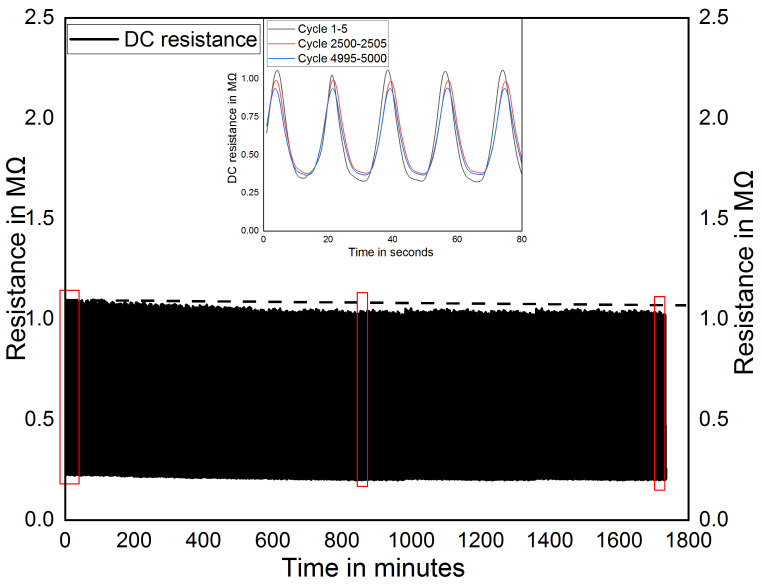
Cyclic response for 5000 cycles.

It was reported that healthy persons would walk around 10,000 steps (which is roughly equivalent to five miles) per day, which means that each foot would be subjected to 5000 steps per day [53]. In order to meet this requirement, the sensor should have a repeatable response around 5000 cycles of pressure loadings. The MWCNT/PDMS pressure sensor is subjected to repeated pressure cycles ranging from 0 to 330 kPa using InstronE10000 universal testing machine. The corresponding DC resistance is measured using Keysight DAQ973A. Figure 6 shows the DC resistance response of the MWCNT/PDMS pressure sensor during 5000 cyclic pressure loading and unloading. It illustrates that the resistance of the sensors exhibited good repeatability with minor fluctuation of maximally 2% relative to the peak resistance of the first cycle. It shows that the MWCNT/PDMS pressure sensor can be used for a long-time foot pressure measurement.

**Figure 7:**
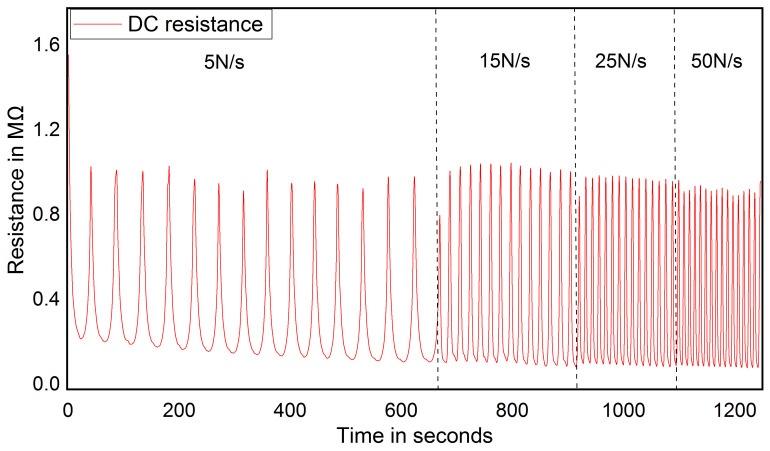
Sensor response on different rate of load.

**Figure 8:**
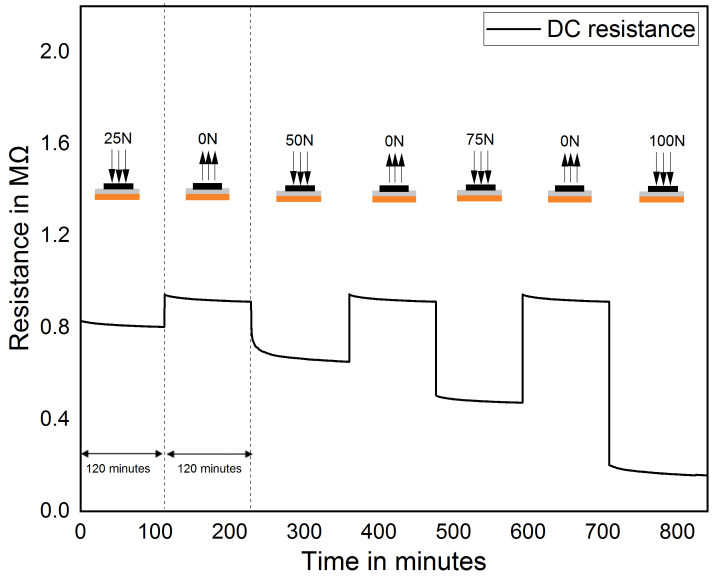
Drift test for MWCNT/PDMS pressure sensor at different pressure applied on it.

One of the essential applications of the proposed foot pressure measurement-based MWCNT/PDMS pressure sensor is rehabilitation monitoring. During a rehabilitation process, the person is subjected to different intensities of physical activities. It stated that in moderate-intensity physical activity, a person walks 90 to 125 steps/min, and jogging of 5 km/hr covers 950 to 1000 steps/min [54,55]. It means that sensors are subjected to a pressure frequency of 5 N/s to 30 N/s. Figure 7 shows sensor response at different pressure frequencies. It shows that the electrical response of the pressure sensor exhibits long-lasting durability with pressure loading rate ranging from 5 N/s to 50 N/s. However, there is a slight fluctuation in the peak value due to the varying contact speed of the load to the sensor. In foot pressure measurement, the sensor should have a stable response under constant pressure loading or unloading over a period of time. This is because an average person can stand in a static position over an extended time. Figure 8 illustrates the electrical response of the sensor under a constant pressure loading at different pressure such as 82 kPa, 117 kPa, 205 kPa and 330 kPa of each cycle. It can be inferred that the sensor resistance becomes stable with a maximum deviation of 0.1% relative to the initial resistance.

### 3.4. Foot Pressure Measurement Using In-Sole Based on MWCNT/PDMS Nanocomposite Pressure Sensor

#### 3.4.1. Preparation of In-Sole Based on MWCNT/PDMS Pressure Sensor

In-sole design and sensor placement mainly depend on foot anatomy like at or high arched foot, clubfoot, and an extra toe. The outcome of Carina et al. showed the pressure parameters in three subareas in the foot such as heel, front foot, and midfoot seems to be measured for an efficient gait analysis [56].

**Figure 9:**
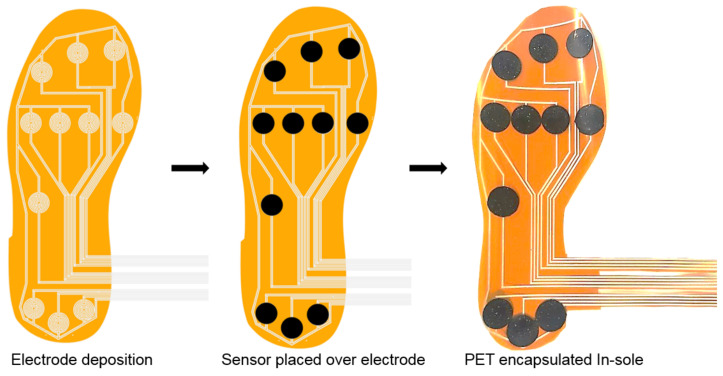
Schematic representation of fabrication of the in-sole with MWCNT/PDMS pressure sensor.

In this work, 11 sensors are distributed in the heel, midfoot and forefoot. In the heel, three sensors are placed. Since pressure distribution is less in the midfoot during the gait and so one sensor is placed. In the forefoot, seven sensors were placed in order to enhance the resolution of foot pressure measurement during the last phase of the gait. Figure 9 shows sensor placement and fabrication steps of the in-sole. The total thickness of the in-sole is 500 μm and weighing approx of 9.46 g.

**Figure 10:**
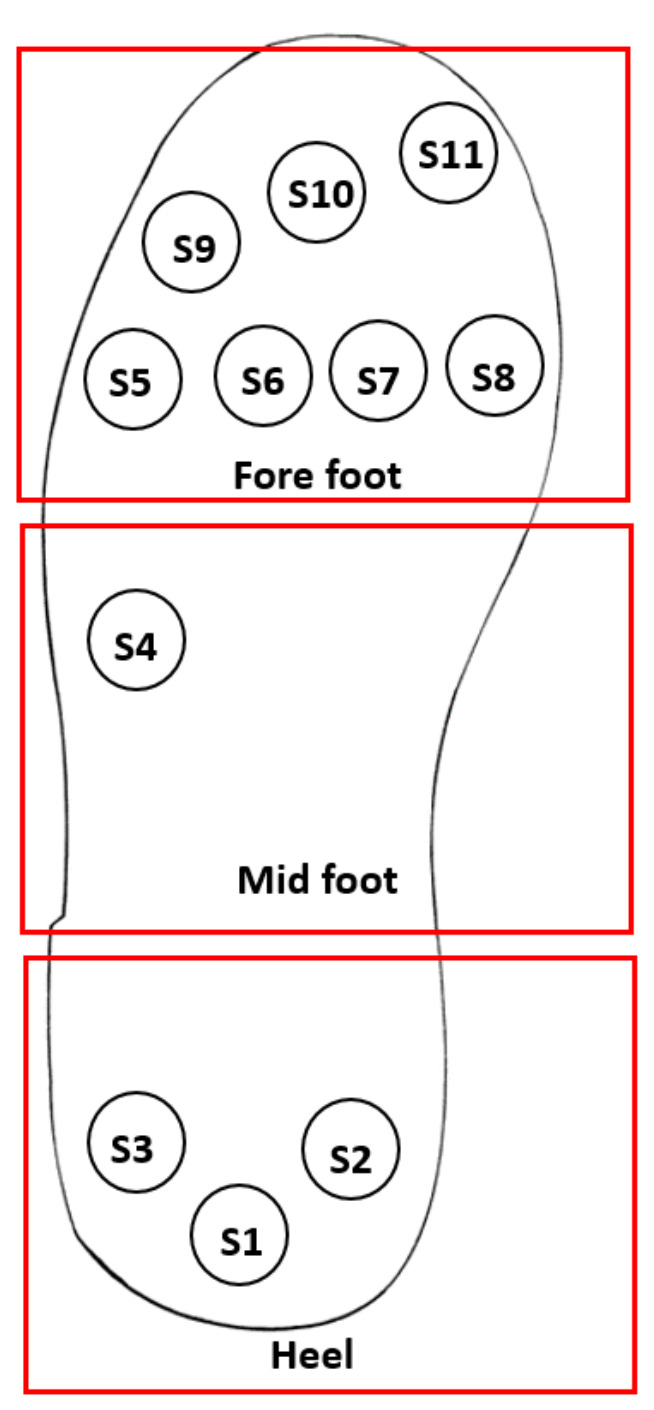
Pressure sensor layout in the sole.

**Figure 11:**
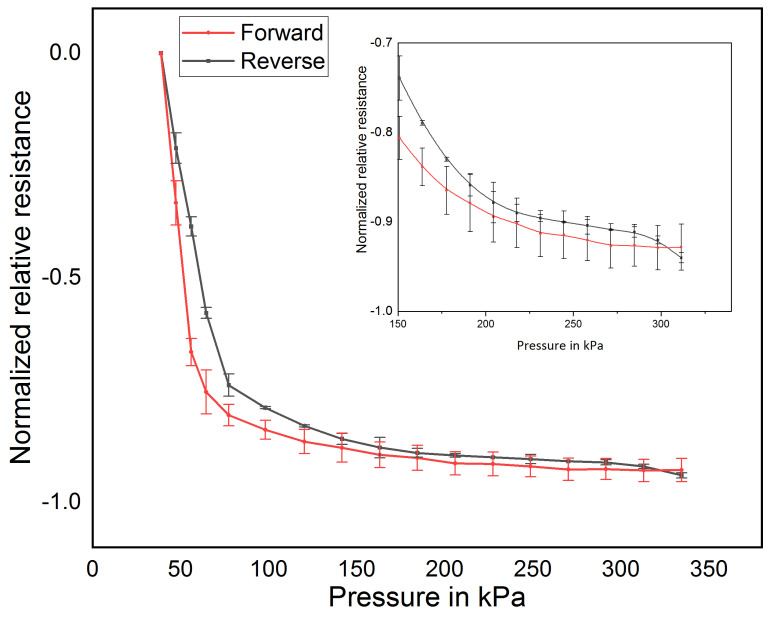
Electro-mechanical behavior of CNT/PDMS pressure sensors implemented in the in-sole.

To test and validate the in-sole system, we first implement a measuring system with the help of an Arduino microcontroller and a voltage divider circuit. Figure 10 indicates the pressure sensor layout and sensor numbers for the gait analysis. Before validating the in-sole for real-time measurements, the electro-mechanical performance for each sensor was characterized. Figure 11 shows the behavior of CNT/PDMS pressure sensors implemented for in-sole applications. It can be seen that the sensor has better reproducible behavior with a maximum deviation of ±2% relative to the normalized relative resistance and the maximum deviation in the sensitivity of ±0.08 kΩ/kPa.

A 60 kg weight of 23 years old healthy female participated in the controlled experiment to validate the system. Pressure distribution on foot was analyzed in stationary and dynamic phases. The in-sole was placed on the ground during the stationary phase, and the person could stand on it. During the Dynamic phase, the in-sole was placed in the right leg of the person’s shoes, and the subject was asked to perform different tests. Foot pressure data was recorded at a time interval of 50ms and then extracted from the database after the experiment for further analysis.

#### 3.4.2. Stationary Phase

**Figure 12:**
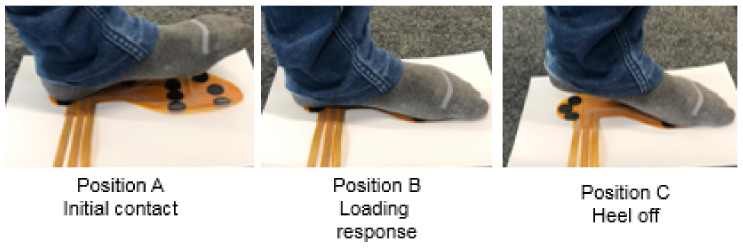
Positions in stationary phase.

**Figure 13:**
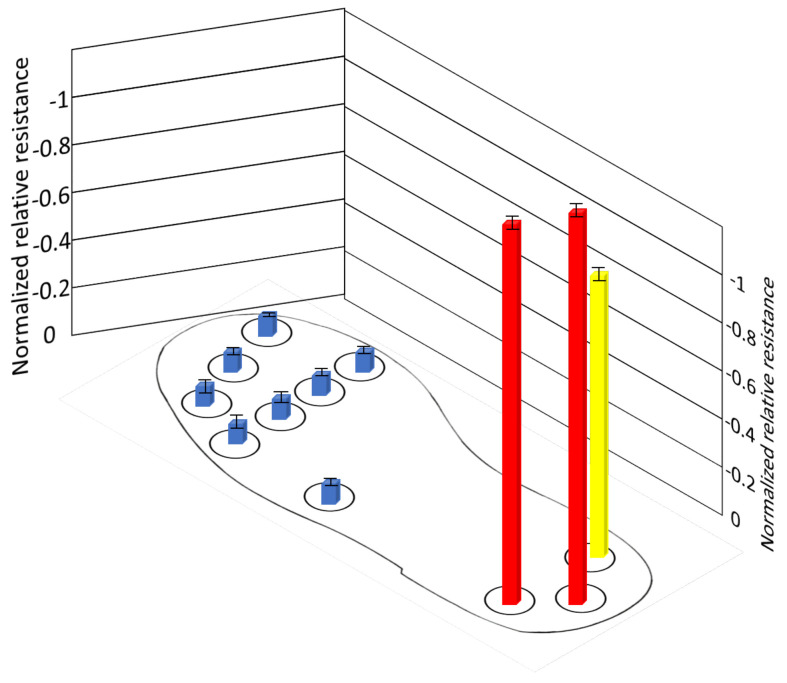
Normalized resistance of sensors during position A.

This experiment investigates the pressure distribution of the different foot sections, such as forefoot, midfoot, and heel, in the stationary scenario. The subject was asked to test each position for 3 s and 5 s gaps in-between the different positions for three cycles to ensure reliable and accurate results. During each position, the person’s leg was taken entirely from the sole and placed again for another position. The higher the relative resistance, the more pressure is applied to it. Figure 12 shows that three positions in the stationary phase, which are defined as follows: Initial contact: when the leg touches the ground and body weight is focused on the heel position. More pressure is exerted on the heel. Loading response: when the leg is completely placed on the ground and the bodyweight is distributed evenly in all three sub-regions. Pressure is evenly distributed on the heel and forefoot. Heel off: when heel lift of from the ground and the body weight is transferred to the forefoot. More pressure is applied on the forefoot. Figure 13 shows the average of normalized resistance for three cycles in position A. It is inferred that the pressure distribution is high in the heel, where normalized resistance of the sensors S1, S2 and S3 are −0.90239 (±0.0045), −0.8097 (±0.0022) and −0.84784 (±0.0037), respectively.

**Figure 14:**
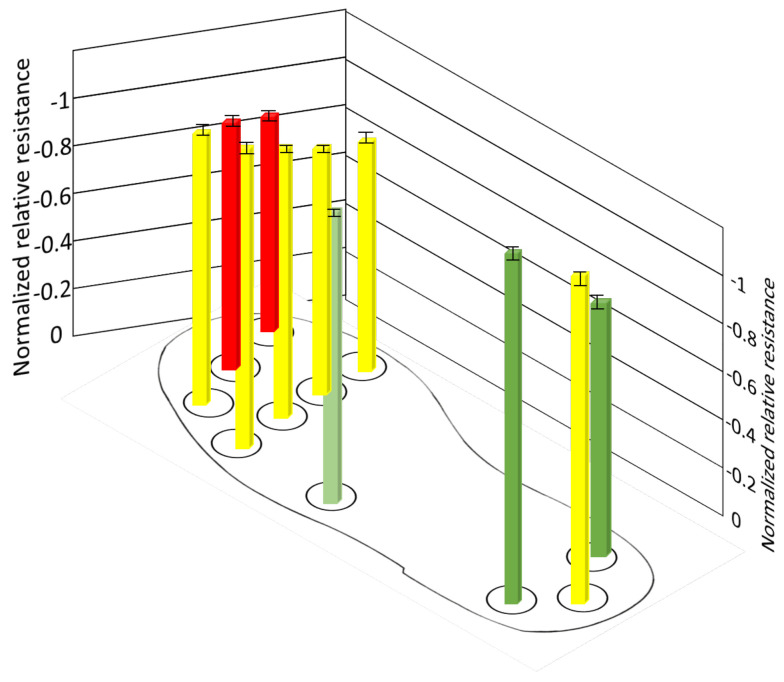
Normalized resistance of sensors during position B.

**Figure 15:**
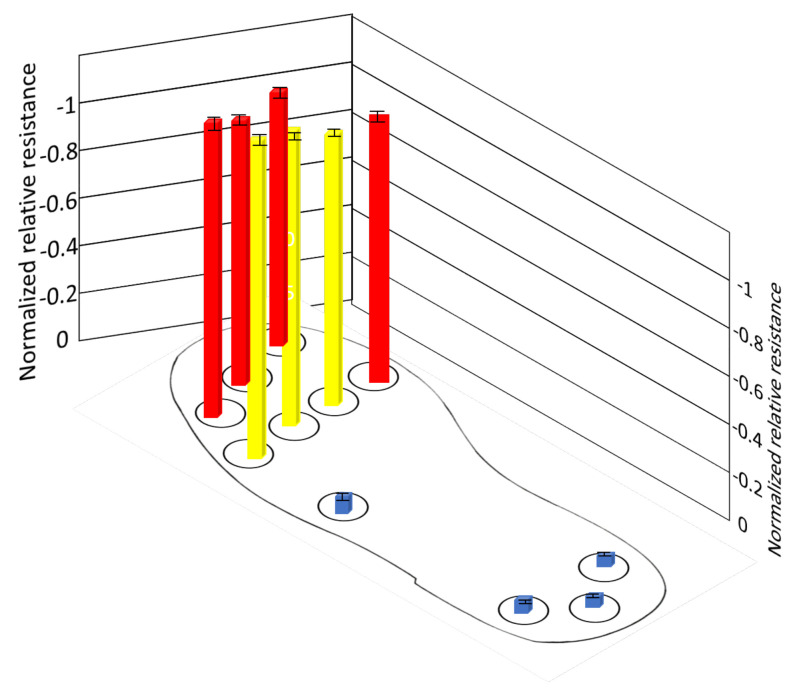
Normalized resistance of sensors during position C.

Which is approximately 160–250 kPa of pressure applied on it. There are no measurable changes in other sensors because those sub-areas are not in contact with the ground. Figure 14 shows the average of normalized resistance for three cycles in position B. The pressure distribution is evenly distributed in all sub-areas, where the normalized resistance of the sensors ranges −0.6083–−0.8091(±0.0045–0.0061). The pressure distribution is approximately 70–90 kPa, 50–60 kPa, and 100–150 kPa in the heel, midfoot, and forefoot, respectively. However, slightly more pressure is applied on the sensor (S9, S10, S11) than other sensors in the forefoot, this might be that the body is slightly moving towards the forward direction in this position. Figure 15 shows the average of normalized resistance for three cycles in position C. The pressure distribution is more in the forefoot, where normalized resistance of the sensors (S5–S11) ranges −0.78283–−0.90316(±0.0025–0.0057), which is approximately 160–250 kPa. There are no measurable changes in sensors in the midfoot and heel because those sub-areas are not in contact with the ground.

#### 3.4.3. Dynamic Phase

**Figure 16:**
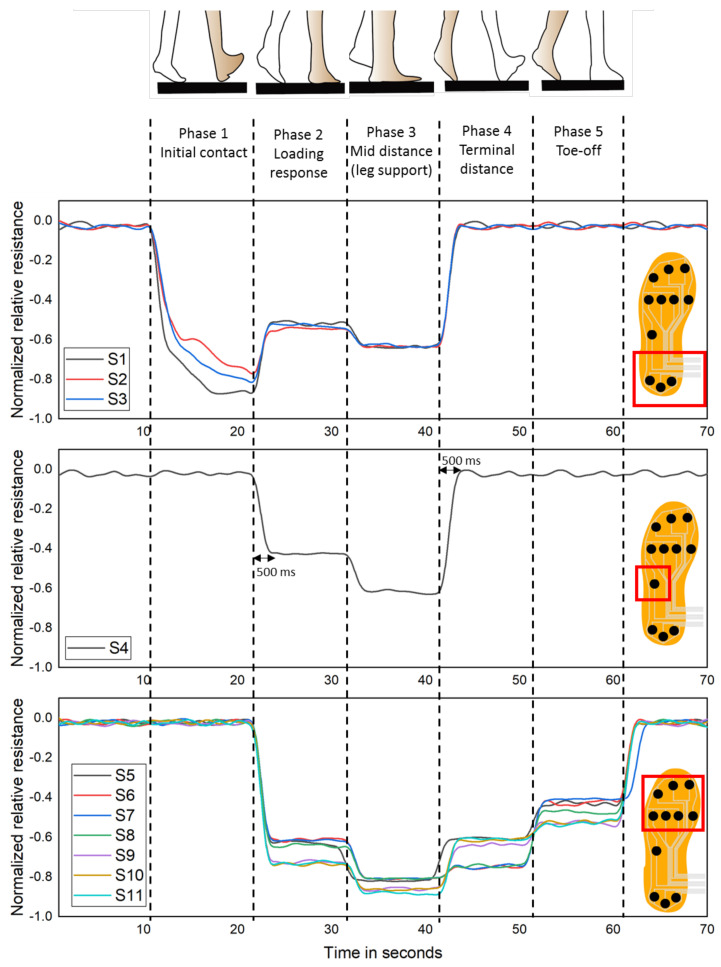
Dynamic response of the pressure sensor during dynamic phase.

This experiment focused on investigating the dynamic gait pattern of a single leg during normal walking, which is considered a swinging leg, where the other one is the supporting leg. The gait pattern is comprised of two stances, and each stance has five steps such as initial contact, load response, mid-distance, terminal distance, and pre-swing. Figure 16 shows that dynamic response of pressure sensors placed on the in-sole when the person is subjected to regular walking, high normalized relative resistance, more pressure is applied on the respective area. The first step of the first instance of normal walking is initial contact, where the body weight is entirely focused on the heel of the swinging leg. So more pressure is applied on the sensor S1–S3, which is in the heel position. However, there is indifference in pressure distribution in S1–S3 because the person is not ideally balanced. The next instance is when the bodyweight is transferred and distributed from the heel to the mid and forefoot of the swinging leg, which can be seen that normalized resistance of S1–S3 is reduced and increased for the S4–S11. Despite the pressure distribution, less pressure is applied on the S4 in the midfoot than other sensors in the forefoot and heel.

When the swinging leg reaches the mid-distance position, the respective foot is subjected to less pressure because the bodyweight is transferred from the supporting foot while it is not in contact with the ground. This makes the slight increase in the normalized resistance of the sensors S1–S11 by 20–30%. In the terminal distance, the heel of the supporting foot and forefoot of the swinging foot contacts the ground, and the pressure is applied on the forefoot (S5–S11). Even though the pressure is applied on the forefoot of the swinging leg, the bodyweight is more transferred to the supporting leg. So the pressure distributed on the forefoot reduced compared to the previous position. In toe-off, only the forefoot in contact with the ground and sensors S5–S11 is subjected to pressure, but the pressure applied on these sensors is still reduced compared to the previous position, where furthermore weight is transferred to the supporting leg. Indeed, it can be seen that the response time of the sensor was around 500 ms when the pressure was applied. This is because the nanocomposite thin films need to contact the electrode during the initial pressure applied.

Table 1 shows the comparison of the proposed sensors with previous works based on CNT/PDMS nanocomposites. It is clearly seen that the proposed sensors have sensitivity, less hysteresis and wide pressure range, compared to the sensors adopted in the previous works with very less concentration of CNT(0.3 wt. %). This demonstrated the efficiency of the proposed sensors for the foot pressure measurement applications.

## 4. Conclusions

In this paper, we introduced a pressuresensor based on multi-walled carbon nanotubes (MWCNT) polydimethylsiloxane (PDMS) with high sensitivity and wide detectable pressure range for gait analysis. We report about the design and fabrication technique of the nanocomposite sensitive material and the pressure sensors achieving low costs and high durability. The characterization of the proposed sensors shows a sensitivity of 4.4 kΩ/kPa, and the hysteresis effect corresponds to an energy loss of less than 1.7% and a measurement deviation of maximally 0.13% relative to the maximal relative resistance, and it can detect a maximum pressure up to 330 kPa. The proposed sensor design meets specific requirements for foot pressure measurement. The resolution and sensor placement can be variable depending on the application of use and highly stable in a robust environment, which lacks commercial FSRs in the sole.

The proposed flexible nanocomposite pressure sensors are then implemented for gait analysis. The analysis of the foot pressure distribution while a person is standing in stationary phase in three-position shows that more pressure distribution in the heel during initial contact, even pressure distribution in both heel and front foot and during toe-off, high-pressure distribution on the front foot. While walking, initially, the pressure distribution is more in the heel and transfer evenly to the midfoot and forefoot during loading response. Then, it transferred entirely to the forefoot during the end of a gait pattern, which is similar to the gait pattern of a healthy person.

## Figures and Tables

**Table 1:** Comparison of proposed in-sole sensor with previous works based on CNT/PDMS pressure sensor for foot pressure measurement.

Author	wt. % of CNT	Sensing Element	Sensitivity	Range	Hyst Eresis (%)	Foot Regions		
						F. f	M. f	Heel
Heng et al. [44]	0.6	6	3.63 MPa${}^{-1}$	25 N	8.9	✓		✓
Wu et al. [45]	-	11	20.9 kPa${}^{-1}$	50 kPa	-	✓	✓	✓
Zhang et al. [23]	5	7	11.5 mV/kPa	265 kPa	12.75	✓		✓
Rajendran et al. [47]	0.3	12	3.312 kΩ/kPa	217 kPa	3.64	✓	✓	✓
**This work**	**0.3**	**11**	**4.4 kΩ** **/kPa**	**330 kpa**	**1.7**	✓	✓	✓

F.f-Fore foot, M.f-Mid foot.

## Data Availability

The data that support the findings of this study are available from the corresponding author, upon reasonable request.

## Abbreviations

The following abbreviations are used in this manuscript:

CNT	carbon nanotubes
PDMS	Polydimethylsiloxane
MWCNT	Multi-walled carbon nanotubes
LD	Linear dichroism
